# Prevalence, Toxigenicity and Antimicrobial Resistance of *Staphylococcus aureus* from Animal-Derived Food Products in the Kostanay Region, Kazakhstan: A Genotypic–Phenotypic Concordance Study

**DOI:** 10.3390/vetsci13070687

**Published:** 2026-07-15

**Authors:** Bakhit Baimenov, Kalamkas Malikzada, Alma Dossova, Zhanaidar Bermukhametov, Pavel Shevchenko, Albina Gabitova, Oxana Tomaruk, Aliya Yskak, Gulzhan Chuzhebayeva, Raushan Rychshanova

**Affiliations:** Research Institute of Innovative Technologies, Kostanay Regional University Named After Akhmet Baitursynuly, Kostanay 110000, Kazakhstan; bahytbajmenov@gmail.com (B.B.); kalamkasmalikzada45@gmail.com (K.M.); dossova.alma95555@gmail.com (A.D.); zhanaidar007@gmail.com (Z.B.); pavel87011339688@gmail.com (P.S.); bibishka03@gmail.com (A.G.); lebleucura@gmail.com (O.T.); aliy.yskak@gmail.com (A.Y.)

**Keywords:** *Staphylococcus aureus*, antimicrobial resistance, staphylococcal enterotoxins, MRSA, BORSA-like phenotype, egc cluster, genotype–phenotype concordance

## Abstract

*Staphylococcus aureus* is a leading cause of foodborne illness in animal-derived food products, yet comprehensive data on its antimicrobial resistance and toxigenic potential from Central Asia remain scarce. We examined 711 food samples (meat, milk, and dairy products) from the Kostanay region of Kazakhstan (2024–2025). Isolates were identified by MALDI-TOF mass spectrometry; antimicrobial susceptibility was assessed by disk diffusion (EUCAST v.16.0); enterotoxin production (types A–E) by ELISA; and resistance and toxin genes by PCR. Concordance between methods was evaluated using Cohen’s kappa coefficient. *S. aureus* was recovered from 11.8% of samples (84/711), with raw cow’s milk carrying the highest contamination risk (OR = 2.75; 95% CI: 1.74–4.38). High resistance to ampicillin (52.4%) and benzylpenicillin (50.0%) was found; 20.2% of strains were multidrug-resistant. Among the 17 cefoxitin-resistant isolates, only 4 harbored mecA, suggesting that 13 isolates (15.5%) should be interpreted as cefoxitin-resistant, mecA-negative isolates with a putative BORSA-like phenotype rather than as confirmed MRSA. Staphylococcal enterotoxins A–E were detected in 26.2% of isolates, and egc-cluster genes seg and sei were present in 9.5% each. The strongest genotype–phenotype concordance was observed for aph(3′)/kanamycin (κ = 0.65) and blaZ/benzylpenicillin (κ = 0.64). These results underscore the need for combined molecular and phenotypic surveillance for regional food safety monitoring.

## Simple Summary

*Staphylococcus aureus* is a bacterium commonly found in animal-derived foods such as milk, meat, and dairy products. It can cause food poisoning by producing heat-resistant toxins and has become increasingly difficult to treat due to antibiotic resistance. This study examined 711 food samples collected across the Kostanay region of Kazakhstan from 2024–2025 to determine how often this bacterium is present, how resistant it is to antibiotics, and whether it carries toxin-producing genes. The bacterium was found in nearly 12% of samples, with raw cow’s milk posing the highest contamination risk. About half of the isolated strains were resistant to commonly used antibiotics, and one in five showed resistance to multiple drug classes. A notable finding was that many strains appeared resistant to an important antibiotic group in standard laboratory tests but lacked the genetic marker typically responsible—a phenomenon that can lead to misidentification. More than a quarter of strains were capable of producing food-poisoning toxins. The results highlight that standard testing alone may be insufficient to accurately assess antibiotic resistance, and that combining genetic and laboratory approaches provides a more reliable picture. These findings provide baseline regional data that may support future food safety monitoring in Kazakhstan and other parts of Central Asia.

## 1. Introduction

*Staphylococcus aureus* is an important foodborne pathogen frequently detected in animal-derived foods, including milk, meat, and dairy products [1,2]. Staphylococcal food poisoning remains a public health concern because *S. aureus* can produce heat-stable enterotoxins that retain biological activity after food processing [1,3,4]. The economic burden of foodborne illness is also substantial: in the United States, it has been estimated at USD 75 billion annually [5], while in developing countries, it exceeds USD 95 billion per year due to productivity losses and healthcare costs [6].

Among the main virulence factors of *S. aureus* are staphylococcal enterotoxins (SEs). Classical enterotoxins A–E are the most commonly investigated in routine food safety testing, while additional enterotoxin genes, including the egc-cluster genes seg and sei, may not be detected by standard commercial ELISA kits [1,7,8,9]. This creates a potential gap between immunological toxin detection and PCR-based gene screening.

Antimicrobial resistance is another important concern. Methicillin-resistant *S. aureus* (MRSA), usually mediated by the mecA gene encoding PBP2a, has been reported in livestock and food products and may enter the food chain through animal production systems [10,11,12,13]. Antibiotic use in livestock can contribute to the selection of multidrug-resistant (MDR) strains, defined as resistance to three or more antimicrobial classes, posing challenges for veterinary and clinical practice [14,15]. However, cefoxitin resistance is not always explained by mecA. Some cefoxitin-resistant, mecA-negative isolates may show a putative BORSA-like phenotype, which has been linked in previous studies to β-lactamase hyperproduction rather than PBP2a-mediated resistance [16,17]. Without molecular confirmation, such isolates may be misinterpreted as true MRSA.

Genotypic and phenotypic methods do not always provide identical results. PCR detects the presence of specific genes, whereas phenotypic tests reflect expressed resistance or toxin production under the conditions used [18]. Therefore, concordance analysis using Cohen’s kappa coefficient can help determine where routine phenotypic testing is reliable and where molecular confirmation is needed [19].

In Kazakhstan, systematic data on *S. aureus* contamination, antimicrobial resistance, and toxigenic potential in animal-derived foods remain limited. This is important because the country has a large livestock sector, antibiotic residues and antimicrobial-resistance risks in animal production have been reported, and raw milk and traditional dairy products are widely consumed [20,21,22,23]. Previous regional studies mainly focused on *S. aureus* from mastitic cow milk [24,25], but did not assess multiple food sources together with genotype–phenotype concordance.

Therefore, the aim of this study was to comprehensively assess the prevalence of *S. aureus* in animal-derived food products from the Kostanay region of the Republic of Kazakhstan, as well as to characterize their antimicrobial resistance and toxigenic potential using phenotypic and molecular genetic methods. In addition, the study aimed to evaluate the concordance between genotypic and phenotypic characteristics and to identify cefoxitin-resistant, mecA-negative isolates with a putative BORSA-like phenotype.

The present study combines the assessment of *S. aureus* prevalence, antimicrobial resistance, enterotoxin production, virulence and resistance genes, and genotype–phenotype concordance within a single regional collection of food-derived isolates.

## 2. Materials and Methods

### 2.1. Study Design, Sampling, and Sample Processing

The study was conducted from January 2024 to March 2025 at the Research Institute of Innovative Technologies of Kostanay Regional University named after Akhmet Baitursynuly (Kostanay, Kazakhstan). Seven categories of animal-derived food products were investigated: beef, poultry meat, horse meat, pork, meat products (sausages, frank-furters), raw cow’s milk, and dairy products (cheese, cottage cheese). A total of 711 samples were examined. Samples were collected from retail markets, grocery chains, and dairy farms across the Kostanay region. The number of samples assigned to each category was not fixed a priori but reflected the relative availability of each product type during field sampling in the Kostanay region. Consequently, raw cow’s milk was represented by a larger number of samples than less frequently available product categories, such as horse meat and pork. All samples were collected under aseptic conditions. Meat samples of at least 100 g and milk samples of at least 100 mL were placed in sterile containers, labeled, and transported to the laboratory under refrigeration at 4 ± 1 °C. Laboratory analysis was initiated no later than 24 h after sample collection.

Solid samples (meat and meat products) (25 g) were homogenized in 225 mL of sterile 0.85% sodium chloride solution (1:10 dilution) using a stomacher for 2 min. Samples of raw cow’s milk and dairy products were diluted 1:10 in sterile 0.85% sodium chloride solution. The prepared suspensions were incubated at 37 °C for 18–24 h before inoculation onto selective media. A qualitative isolation protocol routinely used in our laboratory for the recovery of Staphylococcus aureus from food matrices was applied.

### 2.2. Isolation and Identification of S. aureus

Enriched cultures were plated in parallel onto two selective agars: mannitol salt agar (HiMedia Laboratories Pvt. Ltd., Thane, Maharashtra, India) and CHROMagar Staph aureus (CHROMagar, Paris, France). Plates were incubated at 37 °C for 24–48 h. One to five representative colonies with typical *S. aureus* morphology were selected from each positive sample for further identification, depending on the number of presumptive colonies recovered on the selective medium. Suspicious colonies were subjected to preliminary identification based on a combination of morphological and biochemical characteristics: Gram stain morphology (Gram-positive cocci in grape-like clusters), positive catalase reaction, coagulase activity (rabbit plasma tube test, incubation for 4 h at 37 °C), and DNase activity on DNA agar.

Final species identification was performed by MALDI-TOF mass spectrometry (matrix-assisted laser desorption/ionization-time-of-flight) using the Bruker Biotyper system (Bruker Daltonics GmbH & Co. KG, Bremen, Germany) with database version 3.1. Colonies were prepared by the direct transfer method with an overlay of the α-cyano-4-hydroxycinnamic acid (HCCA) matrix. A score > 2.0 was used as the threshold for reliable species-level identification according to the manufacturer’s criteria [26]. All isolates identified by MALDI-TOF MS as *S. aureus* with a score > 2.0 were additionally confirmed by PCR targeting the nuc gene [24]. Primer sequences, annealing temperatures, and expected amplicon sizes are presented in Table 1.

### 2.3. Antimicrobial Susceptibility Testing

Susceptibility of isolates to 16 antimicrobial agents from six classes was determined by the disk diffusion method (Kirby–Bauer) on Mueller–Hinton agar (HiMedia Laboratories Pvt. Ltd., Thane, Maharashtra, India). The inoculum was prepared from an overnight culture in 0.85% NaCl to a turbidity of 0.5 McFarland units (~1.5 × 10^8^ CFU/mL). Antibiotic disks were placed on the inoculated agar surface and plates were incubated at 35 ± 1 °C for 18–20 h. Inhibition zone diameters were interpreted according to EUCAST clinical breakpoint tables version 16.0 [31] with categorization as susceptible (S), susceptible at increased exposure (I), or resistant (R). *Staphylococcus aureus* ATCC 25923 was used as the quality-control strain for disk diffusion testing. Quality-control zone diameter ranges were interpreted according to CLSI M100 where available; otherwise, manufacturer-recommended QC ranges were used. Clinical isolates were categorized according to EUCAST v16.0 breakpoints where available, independently of the QC strain used. A detailed summary of the disk contents, manufacturers, interpretative standards, zone-diameter breakpoints (S/I/R), and quality-control ranges for each of the 16 antimicrobial agents, including whether EUCAST or manufacturer criteria were applied, is provided in Table S1.

The following agents were tested: β-lactams—ampicillin (10 µg), amoxicillin (25 µg), benzylpenicillin (1 IU), cefoperazone (75 µg), cefoxitin (30 µg); tetracyclines—tetracycline (30 µg), doxycycline (30 µg); macrolides—erythromycin (15 µg), tylosin (30 µg); aminoglycosides—streptomycin (10 µg), kanamycin (30 µg), neomycin (30 µg), gentamicin (10 µg); sulfonamides—sulfamethoxazole/trimethoprim (1.25/23.75 µg); fluoroquinolones—ciprofloxacin (5 µg), norfloxacin (10 µg). Cefoxitin was used as a surrogate marker for methicillin resistance in accordance with the EUCAST recommendations [31]. Multidrug resistance (MDR) was defined as acquired resistance to three or more antimicrobial classes according to the criteria of Magiorakos et al. [15].

### 2.4. Toxigenicity Testing and PCR Genotyping

Toxigenic properties of isolates were assessed in two stages. In the first stage, production of staphylococcal enterotoxins (SEs) of types A–E was detected by enzyme-linked immunosorbent assay (ELISA) using the RIDASCREEN SET Total Kit (R-Biopharm AG, Darmstadt, Germany), validated in accordance with international standard EN ISO 19020 [32]. The method is based on a sandwich ELISA principle with polyclonal antibodies specific to SE types A–E; the analytical sensitivity of the kit is 0.1–0.2 ng/mL for each detected toxin. Assays were performed strictly according to the manufacturer’s instructions; results were evaluated photometrically at 450 nm. An isolate was considered SE-positive when the optical density exceeded the cut-off value established by the manufacturer.

In the second stage, all isolates—regardless of ELISA result—were subjected to PCR-based identification of resistance genes for β-lactams (blaZ, mecA), macrolides (ermC, msrA), aminoglycosides (aac(6′)-aph(2″), aph(3′)), tetracyclines (tet(K), tetM), and sulfamethoxazole/trimethoprim (dfrG), as well as enterotoxin genes sea, seb, sec, sed, see, seg, and sei. The PCR methodology is described in Section 2.5. Parallel genotyping of toxigenicity and resistance genes enabled the detection of discordances between genotypic and phenotypic data. Primer sequences and amplicon sizes are presented in Table 1.

### 2.5. Detection of Antimicrobial Resistance and Enterotoxin Genes by PCR

Genomic DNA was extracted by thermal lysis: a cell suspension in 200 µL of sterile distilled water was heated at 95 °C for 10 min, followed by centrifugation at 13,000 rpm for 5 min [33]. The supernatant was used directly as the DNA template. PCR was performed in a total volume of 25 µL containing 12.5 µL DreamTaq Green Master Mix (Thermo Fisher Scientific, Waltham, MA, USA), 10 pmol of each primer, and 1.5 µL of DNA template. All PCR assays were performed as singleplex reactions, with a single primer pair amplified per reaction. Amplification conditions were as follows: initial denaturation at 94 °C for 5 min; 30 cycles of denaturation at 94 °C for 30 s, annealing at 45–61 °C for 1 min (temperature varied by primer pair, see Table 1), and extension at 72 °C for 1 min; final extension at 72 °C for 10 min. Positive-control DNA for species confirmation was obtained from the reference *Staphylococcus aureus* strain ATCC^®^ 25923™ (American Type Culture Collection, Manassas, VA, USA). Sterile nuclease-free water was used as the negative control in each PCR run. PCR assays were performed once for each isolate, while samples with ambiguous or weak amplification bands were retested to confirm the result. Gene-specific positive controls were not available for antimicrobial resistance and entero-toxin genes. Therefore, primer sets previously validated in published studies were used [25,27,28,29,30], and amplification products were verified by their expected fragment sizes. Samples with ambiguous amplification patterns were retested. PCR products were separated by electrophoresis in 1.5% agarose gel at 100 V for 30 min, visualized under UV illumination, and identified according to their expected fragment sizes (Table 1).

### 2.6. Statistical Analysis

Concordance between genotypic and phenotypic methods was assessed using Cohen’s kappa coefficient (κ) with the following interpretation scale: ≤0—no agreement; 0.01–0.20—slight; 0.21–0.40—fair; 0.41–0.60—moderate; 0.61–0.80—substantial; 0.81–1.00—almost perfect [19]. Diagnostic performance of each genotypic marker relative to the corresponding phenotypic result was calculated from 2 × 2 contingency tables, including sensitivity (Se), specificity (Sp), positive predictive value (PPV), and negative predictive value (NPV) [34].

Variation in contamination rates across sample sources was evaluated using Pearson’s chi-squared test; when significant differences were detected, post hoc analysis was performed using Fisher’s exact test with Bonferroni correction (adjusted significance threshold α = 0.05/7 = 0.007) [35,36]. Odds ratios (OR) with 95% confidence intervals (CI) were calculated for each source using Woolf’s method [37]. Associations between specific resistance genes and the MDR phenotype were assessed by Fisher’s exact test with calculation of OR and 95% CI [35,37]. Multivariable logistic regression was applied to identify independent predictors of the MDR phenotype, with all significant variables entered simultaneously [38]. Statistical significance was set at *p* < 0.05 (two-tailed). All analyses were performed in Python 3.11 using the scipy v.1.11 and numpy v.1.26 libraries [39,40]. Antimicrobial resistance profiles and gene distribution were visualized using heatmaps and hierarchical clustering generated with the ClustVis web platform [41], applying Manhattan distance and average linkage methods.

## 3. Results

### 3.1. Prevalence of S. aureus and Source-Specific Contamination Risk

*S. aureus* was isolated from 84 out of 711 animal-derived food samples (11.8%). The isolation rate differed significantly across the seven product categories (χ^2^ = 20.19; df = 6; *p* = 0.003). The highest prevalence was observed in raw cow’s milk samples—19.2% (47/245)—while among meat products, it ranged from 4.3% (horse meat, 1/23) to 10.0% (pork, 2/20).

Post hoc analysis with Bonferroni correction (α = 0.05/7 = 0.007) identified raw cow’s milk as the only source with a significantly elevated contamination risk: OR = 2.75 (95% CI: 1.74–4.38; *p*_corr_ < 0.001). No statistically significant differences relative to the overall contamination rate (11.8%) were found for any other product category (all *p*_corr_ ≥ 0.697). Detailed prevalence and contamination risk data for all sources are presented in Table 2.

The number of samples differed across product categories. Consequently, categories represented by fewer samples, such as horse meat and pork, showed wider 95% confidence intervals, indicating greater uncertainty of the corresponding prevalence estimates.

**Figure 1:**
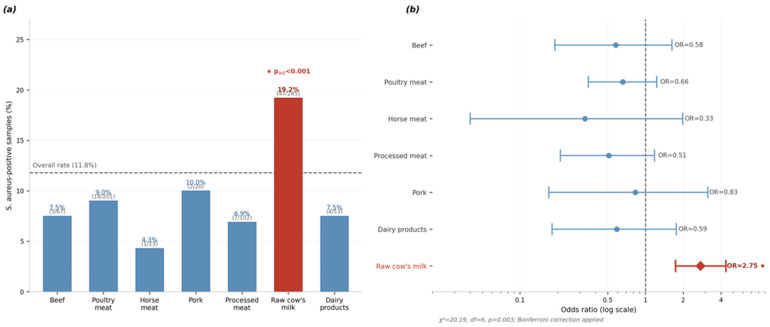
Prevalence of *S. aureus* across seven animal-derived food product categories and source-specific contamination risk estimates. (**a**) Proportion of *S. aureus*-positive samples (%) per source; the dashed horizontal line indicates the overall contamination rate (11.8%). (**b**) Forest plot of odds ratios (ORs) with 95% confidence intervals for each source versus all others combined; the dashed vertical line at OR = 1 indicates no difference from the overall rate. ★ *p*_corr_ < 0.001; Bonferroni correction applied.

The distribution of contamination rates across sources and the corresponding OR estimates with 95% CIs are illustrated in Figure 1, highlighting the disproportionate contribution of raw cow’s milk to the overall contamination burden.

As shown in Figure 1, contamination rates varied considerably across the seven food product categories. Raw cow’s milk exhibited the highest *S. aureus* prevalence (19.2%; 47/245) and was the only source with an odds ratio significantly above 1 (OR = 2.75; 95% CI: 1.74–4.38), indicating a nearly 2.75-fold higher contamination risk compared to all other sources combined. All remaining categories—beef (7.5%), poultry meat (9.0%), horse meat (4.3%), pork (10.0%), processed meat (6.9%), and dairy products (7.5%)—showed contamination rates at or below the overall average and odds ratios below 1, none of which reached statistical significance after Bonferroni correction (all *p*_corr_ ≥ 0.697).

### 3.2. Antimicrobial Susceptibility

Assessment of susceptibility of the 84 isolates to 16 antimicrobial agents from six classes revealed the highest resistance rates among β-lactams: 52.4% of isolates were resistant to ampicillin and 50.0% to benzylpenicillin (Table 3).

Cefoxitin resistance was detected in 20.2% of isolates (17/84); molecular characterization of this group is presented in Section 3.6. Among the tetracyclines, resistance to tetracycline (40.5%) was more than twice that to doxycycline (19.0%). Macrolide resistance rates were 32.1% for erythromycin and 26.2% for tylosin. Aminoglycosides and sulfamethoxazole/trimethoprim retained high activity, with susceptible isolates exceeding 86.9% for all agents in these classes. All 84 isolates were susceptible to streptomycin. For ciprofloxacin, 75 isolates (89.3%) fell between the resistant breakpoint and the off-scale susceptible breakpoint used by EUCAST for Staphylococcus aureus; these isolates were not interpreted as fluoroquinolone-resistant and were not counted as resistant in MDR classification.

### 3.3. Multidrug Resistance

In terms of the number of antibiotics to which resistance was detected, 25.0% of isolates were fully susceptible, while 19.0% showed resistance to five or more agents (Table 4).

By the MDR criterion (resistance to three or more antimicrobial classes), 17 isolates (20.2%) were classified as multidrug-resistant (Table 5 and Table S2). MDR was classified by antimicrobial class rather than individual agent, which explains the discrepancy between Table 4 and Table 5.

**Figure 2:**
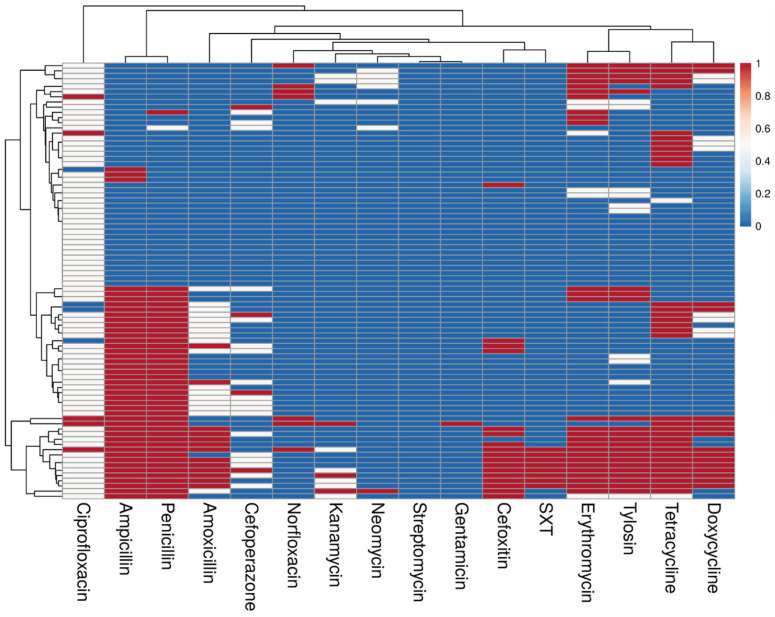
Hierarchical heatmap of antimicrobial resistance profiles of 84 *S. aureus* isolates. Red indicates resistant isolates (R = 1), white indicates intermediate susceptibility (I = 0.5), and blue indicates susceptible isolates (S = 0). Clustering was performed using Manhattan distance and average linkage.

Co-resistance patterns were visualized by hierarchical clustering in a heatmap (Figure 2).

Most isolates were susceptible to aminoglycosides and norfloxacin, while resistance was predominantly observed to β-lactams, macrolides, and tetracycline. A high proportion of isolates fell between the resistant breakpoint and the off-scale susceptible breakpoint for ciprofloxacin. Clustering revealed similar resistance profiles between ampicillin and benzylpenicillin, erythromycin and tylosin, and tetracycline and doxycycline, suggesting shared resistance mechanisms.

**Figure 3:**
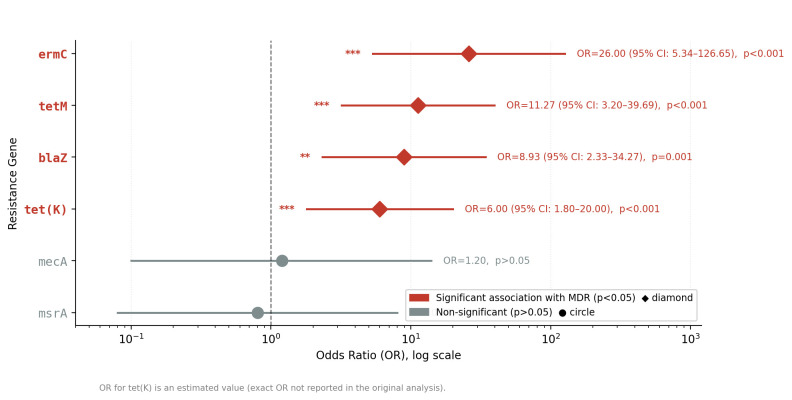
Forest plot of associations between resistance genes and the MDR phenotype (Fisher’s exact test; log-scale OR). Significant associations (*p* < 0.05): ermC (OR = 26.00; 95% CI: 5.34–126.65), tetM (OR = 11.27; 95% CI: 3.20–39.69), blaZ (OR = 8.93; 95% CI: 2.33–34.27), tet(K) (*p* < 0.001)—red diamonds (◆). Non-significant associations (*p* > 0.05): mecA, msrA—grey circles (●). *** *p* < 0.001; ** *p* < 0.01.

Fisher’s exact test identified significant associations with the MDR phenotype for ermC (OR = 26.00; 95% CI: 5.34–126.65; *p* < 0.001), tetM (OR = 11.27; 95% CI: 3.20–39.69; *p* < 0.001), blaZ (OR = 8.93; 95% CI: 2.33–34.27; *p* = 0.001), and tet(K) (*p* < 0.001). The mecA and msrA genes showed no significant association with MDR (both *p* > 0.05). These results are presented in Figure 3.

### 3.4. Toxigenic Potential

ELISA detected the production of at least one SE types A–E in 22 out of 84 isolates (26.2%), with a total of 47 positive reactions accounting for the co-production of multiple toxins by individual isolates. SEE (14.3% of isolates) and SED (13.1%) predominated, while SEA was detected in 10.7% of isolates. Among the SE-positive isolates, monotoxigenic strains predominated (50.0%); however, 13.6% of SE-positive isolates tested positive for all five classical SE types (A–E) by ELISA (Table 6).

The highest proportion of SE-positive isolates was observed in dairy products (75.0%; 3/4), although this result should be interpreted with caution given the small number of isolates in this group. In raw cow’s milk and non-dairy products, the SE-positive rate was 29.8% and 21.6%, respectively. The overall SE-positive rate among all *S. aureus* isolates was 26.2%. These findings suggest a trend toward higher prevalence of enterotoxigenic strains in dairy products.

### 3.5. PCR Genotyping of Resistance and Toxigenicity Genes

PCR genotyping of 84 isolates revealed substantial variability in the detection frequency of individual resistance and toxigenicity genes (Table 7).

The most prevalent resistance gene was tet(K), detected in 61.9% of isolates—nearly twice the frequency of tetM (33.3%). blaZ was found in 44.0% of isolates and ermC in 35.7%. mecA, aac(6′)-aph(2″), aph(3′), and dfrG were detected infrequently (3.6–4.8%). The most common gene combinations were tet(K) + ermC (29.8%), tet(K) + tetM (28.6%), and tet(K) + blaZ (27.4%); the four-gene combination blaZ + tet(K) + tetM + ermC was found in 13.1% of isolates.

**Figure 4:**
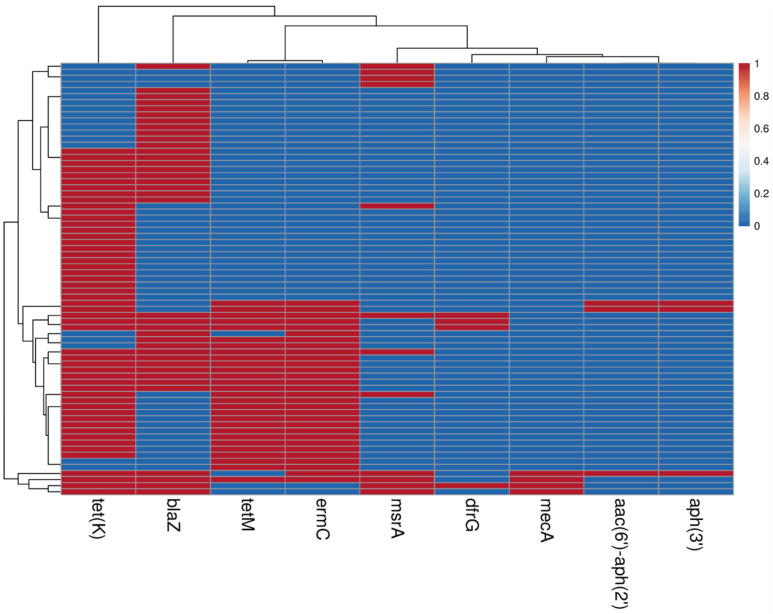
Hierarchical heatmap of antimicrobial resistance gene distribution among 84 *S. aureus* isolates. The heatmap is based on a binary presence (1)/absence (0) matrix. Red indicates gene presence (1), blue indicates gene absence (0). Clustering was performed using Manhattan distance and average linkage.

Among the toxigenicity genes, seg and sei predominated (9.5% each), always co-detected. The genes sea, seb, sec, and see were found in only sporadic isolates (≤4.8%), and sed was not detected in any isolate. Resistance gene distribution patterns are visualized in Figure 4.

Hierarchical clustering of resistance genes revealed marked heterogeneity in genetic profiles among the isolates (Figure 4). The most prevalent genes—tet(K), blaZ, tetM, and ermC—formed the primary genetic profiles of isolates with multiple resistance determinants, while mecA, dfrG, aac(6′)-aph(2″), and aph(3′) were detected in only a limited number of strains. Cluster analysis also demonstrated the co-occurrence of tetM and ermC, and of tet(K) with blaZ, indicating the spread of combined resistance mechanisms among circulating isolates.

Among the toxigenicity genes, seg and sei were most frequently detected, while classical enterotoxin genes sea, seb, sec, and see were found only sporadically, and sed was absent. In several isolates, resistance and toxigenicity genes co-occurred—including combinations of tet(K), tetM, ermC, and blaZ with seg/sei—suggesting the co-occurrence of resistance and virulence determinants among food isolates of *S. aureus*.

### 3.6. Cefoxitin Resistance and Putative BORSA-like Phenotype

**Figure 5:**
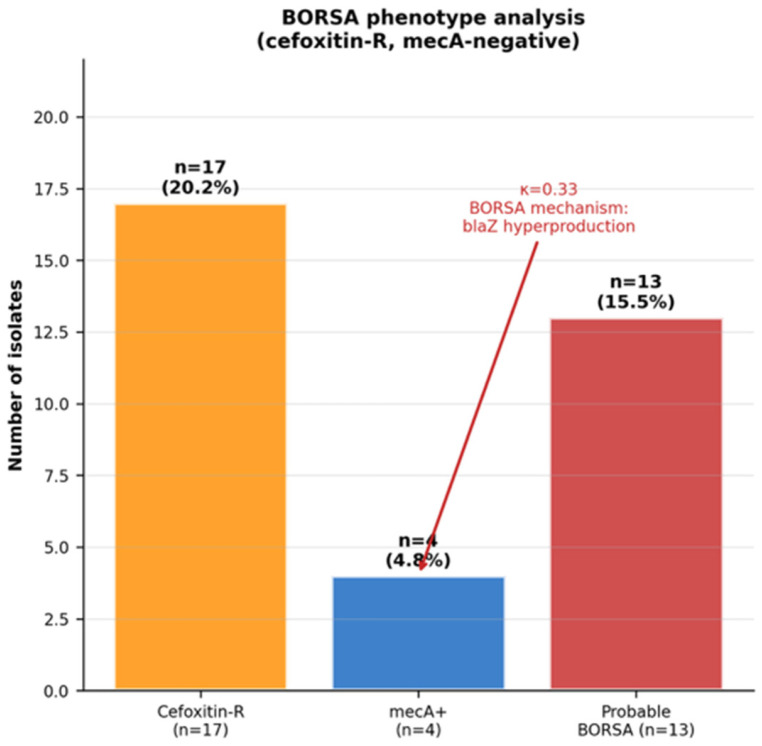
Distribution of cefoxitin-resistant *S. aureus* isolates, mecA-positive MRSA, and isolates with a putative BORSA-like phenotype. The putative BORSA-like phenotype was defined as cefoxitin resistance in the absence of mecA; definitive confirmation would require oxacillin MIC testing, assessment of β-lactamase hyperproduction, mecC screening, and/or PBP2a testing.

Despite the cefoxitin-resistant phenotype observed in a subset of isolates, molecular analysis revealed a limited distribution of mecA. Of the 17 cefoxitin-resistant isolates (20.2%), only 4 (4.8%) carried mecA by PCR. Accordingly, 13 isolates (15.5%) were interpreted as cefoxitin-resistant, mecA-negative isolates with a putative BORSA-like phenotype. Definitive BORSA confirmation would require oxacillin MIC determination, β-lactamase hyperproduction testing, mecC screening, and/or PBP2a testing, which were not performed in this study. The blaZ detection rate among these isolates (53.8%; 7/13) suggests a possible β-lactamase-mediated mechanism in a subset of isolates, but this remains presumptive. Concordance between cefoxitin phenotypic resistance and mecA carriage was poor (κ = 0.33), with a sensitivity of 23.5% and specificity of 100%. The distribution of cefoxitin-resistant, mecA-positive, and putative BORSA-like isolates is shown in Figure 5. Individual isolate-level MRSA/BORSA-like phenotypes are provided in Table S2.

The bar chart shows the distribution of *S. aureus* isolates within the cefoxitin-resistant subset: all cefoxitin-R isolates (orange, n = 17; 20.2% of 84), mecA-positive true MRSA (blue, n = 4; 4.8%), and putative BORSA were cefoxitin-resistant in the absence of mecA (red, n = 13; 15.5%). The annotation indicates fair concordance between cefoxitin resistance and mecA carriage (κ = 0.33) and a possible blaZ-associated mechanism, which remains presumptive because β-lactamase hyperproduction was not directly assessed. All percentages are expressed relative to the total isolate collection (n = 84).

### 3.7. Genotypic–Phenotypic Concordance

**Figure 6:**
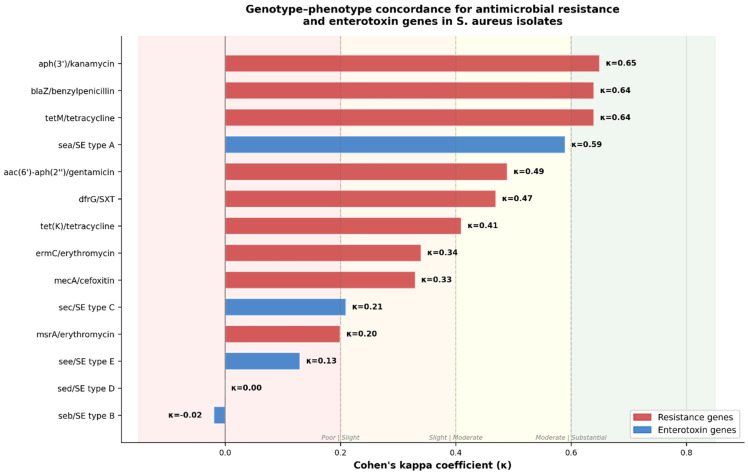
Concordance between genotypic and phenotypic methods for antimicrobial resistance and toxigenicity in *S. aureus* (Cohen’s kappa coefficient, κ). Red—resistance genes; blue—enterotoxin genes. Vertical dashed lines indicate interpretation thresholds: ≤0—no agreement; 0.01–0.20—slight; 0.21–0.40—fair; 0.41–0.60—moderate; 0.61–0.80—substantial; 0.81–1.00—almost perfect. Positive κ values indicate the degree of concordance between genotypic and phenotypic methods.

Comparison of PCR results with disk diffusion and ELISA data revealed a wide range of Cohen’s kappa values (κ = −0.02 to 0.65), reflecting varying degrees of concordance between genotypic and phenotypic methods (Table 8 and Table S3, Figure 6).

Diagnostic performance estimates for markers with rare or absent gene detection (e.g., seb, sed) should be interpreted with caution: small numerator counts, and for sed, the complete absence of PCR-positive isolates, produce unstable point estimates (PPV not calculable) and near-zero or negative kappa values that do not reliably reflect true diagnostic accuracy.

Substantial concordance (κ ≥ 0.60) was established for aph(3′)/kanamycin (κ = 0.65), blaZ/benzylpenicillin (κ = 0.64), and tetM/tetracycline (κ = 0.64). Moderate concordance was observed for aac(6′)-aph(2″)/gentamicin (κ = 0.49), dfrG/sulfamethoxazole–trimethoprim (κ = 0.47), tet(K)/tetracycline (κ = 0.41), and sea/SEA (κ = 0.59). Fair concordance was found for ermC/erythromycin (κ = 0.34), mecA/cefoxitin (κ = 0.33), msrA/erythromycin (κ = 0.20), sec/SEC (κ = 0.21), and see/SEE (κ = 0.13).

The lowest kappa values were observed for seb/SEB (κ = −0.02) and sed/SED (κ = 0.00). Notably, sed was not detected in any isolate by PCR, while SED production was detected by ELISA in 11 isolates (13.1%). Overall, enterotoxin genes showed lower genotypic–phenotypic concordance than resistance genes. Results are presented in Figure 6.

Multivariable logistic regression including the four genes significantly associated with MDR in univariable analysis (blaZ, tetM, ermC, tet(K)) revealed the loss of individual statistical significance for each predictor in the multivariable model (all *p* > 0.05). All four variables were entered simultaneously, without a formal stepwise or penalized selection procedure or formal goodness-of-fit assessment. The variance inflation factor (VIF) exceeded 5.0 for all four variables, confirming pronounced multicollinearity; therefore, the resulting coefficient estimates should be interpreted with caution. Given this multicollinearity and the relatively limited number of MDR-positive isolates available for multivariable modeling, these regression results should be interpreted as descriptive of co-occurrence among resistance genes rather than as evidence for independent, causally interpretable predictors of the MDR phenotype; the univariable Fisher’s exact test associations reported above remain the primary evidence base for gene-level associations in this study.

### 3.8. Distribution of SE-Positive Isolates by Source

Analysis of the distribution of SE-positive isolates by sample source revealed marked variability in toxigenic strain prevalence, ranging from 0% in horse meat to 75.0% in dairy products (cheese and cottage cheese). A trend toward a higher prevalence of enterotoxigenic strains was observed in dairy products (OR = 9.63; 95% CI: 0.88–104.8), although the difference did not reach statistical significance. In raw cow’s milk, the SE-positive rate was 29.8%, slightly above the overall sample mean (26.2%; *p* = 0.460).

## 4. Discussion

This study was designed as a regional cross-sectional survey rather than a stratified or establishment-level sampling scheme. Sample numbers were not allocated according to a pre-specified power calculation but reflected the availability of animal-derived food products during field sampling in the Kostanay region. This should be considered when interpreting source-specific prevalence estimates and odds ratios, particularly for less-represented categories such as pork and horse meat, where smaller sample sizes resulted in wider confidence intervals and lower precision.

The overall prevalence of *S. aureus* was 11.8%, which is lower than many published estimates, including pooled rates of 21.7% in meat and 29.2% in raw meat products [2,42]. Raw cow’s milk showed the highest contamination rate (OR = 2.75; 95% CI: 1.74–4.38), consistent with the recognized role of bovine mastitis, milking hygiene, and cold-chain conditions in dairy contamination [22,23]. Together with earlier regional studies [24,25], these findings suggest recurrent, but not continuous, detection of *S. aureus* in dairy-related samples from the Kostanay region during 2020–2025. In contrast, contamination rates in meat categories were lower than those reported in several global studies [43,44,45].

Resistance was mainly observed to β-lactams, tetracyclines, and macrolides. The high resistance to ampicillin and benzylpenicillin is consistent with the frequency of blaZ and with global data on dairy-derived *S. aureus* [46]. Tetracycline and macrolide resistance may also be consistent with the veterinary use of these antimicrobial classes, although antibiotic-use data were not collected in this study. Frank ciprofloxacin resistance was rare, while most isolates fell between the resistant breakpoint and the off-scale susceptible breakpoint used by EUCAST for Staphylococcus aureus. These isolates were not interpreted as fluoroquinolone-resistant, but this pattern may still be useful to monitor in food-chain surveillance. The MDR rate was 20.2% and should be interpreted descriptively because antibiotic use, regulatory enforcement, and farm-level management were not assessed.

Cefoxitin resistance should be distinguished from confirmed methicillin resistance. Although 20.2% of isolates were cefoxitin-resistant, only 4.8% carried mecA, a value comparable to the overall pooled global MRSA prevalence reported for meat and meat products [10]. The remaining 13 cefoxitin-resistant, mecA-negative isolates were interpreted as having a putative BORSA-like phenotype rather than confirmed BORSA. blaZ was detected in 7 out of these 13 isolates (53.8%), suggesting a possible β-lactamase-mediated explanation in some isolates, consistent with previous reports linking such phenotypes to β-lactamase hyperproduction [16]. However, β-lactamase hyperproduction, oxacillin MIC, PBP2a, and mecC were not assessed; therefore, this mechanism remains presumptive, and some isolates could theoretically represent undetected mecC-positive MRSA. The frequency of cefoxitin-resistant, mecA-negative isolates with a putative BORSA-like phenotype (15.5%) exceeded the global food-product estimate of 8.9% [47], further sup-porting the need for molecular confirmation of mecA, and ideally, mecC when cefoxitin-resistant food isolates are detected.

The predominance of tet(K) over tetM is consistent with plasmid-borne tetracycline resistance in *S. aureus* [48,49] and with earlier regional findings [24,25]. The genes ermC, tetM, blaZ, and tet(K) were associated with MDR in univariable analysis. However, multivariable regression showed pronounced multicollinearity, with VIF values exceeding 5.0 for all four variables. These results therefore indicate the co-occurrence of resistance genes rather than independent predictors of MDR. Confirmation of possible co-mobilization would require plasmid profiling or whole-genome sequencing.

The toxigenicity results highlight the importance of interpreting ELISA and PCR data together. ELISA detected at least one classical enterotoxin A–E in 26.2% of isolates, with SED and SEE predominating over SEA, a pattern consistent with milk- and dairy-associated *S. aureus* populations [30]. However, PCR did not fully support the ELISA findings. The sed gene was not detected in any isolate despite SED positivity by ELISA, while see was found in only one isolate despite frequent SEE positivity. Similarly, the apparent A/B/C/D/E ELISA profile observed in 13.6% of SE-positive isolates was not supported by PCR, as sea, seb, sec, sed, and see were detected in only 4, 1, 1, 0, and 1 isolates, respectively. These discrepancies may reflect cross-reactivity of the polyclonal antibodies used in the ELISA kit with structurally related enterotoxins, divergent toxin gene alleles not detected by the primers used, or other assay-related limitations. Further clarification would require expanded PCR panels and protein-level confirmation, for example, by mass spectrometry. The co-detection of seg and sei in 9.5% of isolates also indicates a limitation of routine toxin screening, because standard ELISA kits for classical enterotoxins A–E do not detect egc-cluster proteins. Although egc-cluster enterotoxins have been implicated in food-poisoning investigations [9], their contribution to food safety risk in this study remains uncertain because they were detected only at the DNA level.

Overall, genotype–phenotype concordance was strongest for direct gene–antibiotic pairs, including aph(3′)/kanamycin, blaZ/benzylpenicillin, and tetM/tetracycline. Lower agreement for mecA/cefoxitin, sed/SED, and see/SEE shows that phenotypic or molecular testing alone may be insufficient for the reliable characterization of food-derived *S. aureus*. Combining both approaches therefore provides a more reliable basis for regional food safety surveillance.

The present study has several limitations. It was conducted in a single region of Kazakhstan and used a cross-sectional design, limiting national extrapolation and preventing the assessment of temporal trends. Whole-genome sequencing, MLST, spa typing, and phylogenetic analysis were not performed; therefore, clonal lineages, sequence types, spa types, transmission relationships, and the distribution of mobile genetic elements could not be determined. Finally, the multivariable regression analysis should be considered exploratory because of the limited number of MDR isolates, multicollinearity, and the absence of formal goodness-of-fit diagnostics.

## 5. Conclusions

This regional cross-sectional study provides a comprehensive characterization of the prevalence, toxigenic potential, and antimicrobial resistance of *Staphylococcus aureus* isolated from animal-derived food products in the Kostanay region of Kazakhstan. Raw cow’s milk showed the highest contamination rate among the investigated food categories, highlighting the importance of strengthened microbiological monitoring of this product group.

The findings demonstrate that phenotypic testing alone may be insufficient for the reliable characterization of selected *S. aureus* traits. This was particularly evident for cefoxitin resistance and enterotoxin detection, where notable discrepancies were observed between the phenotypic and molecular results. These observations support the value of integrating phenotypic and molecular approaches to improve the accuracy of antimicrobial resistance and toxigenicity surveillance in food-derived *S. aureus*.

Although the study was limited to a single region and did not include the molecular typing of isolates, it expands the currently available data on food-derived *S. aureus* from Central Asia and provides a basis for future investigations incorporating whole-genome sequencing and molecular epidemiological approaches. The findings may help inform future regional food safety surveillance programs and antimicrobial resistance monitoring strategies.

## Figures and Tables

**Table 1:** Primers used for the identification of Staphylococcus aureus and detection of antimicrobial resistance and enterotoxin genes.

Gene	Target/Function	F/R	Sequence 5′–3′	Ta (°C)	bp	Reference
nuc	*S. aureus* species-specific thermonuclease (species identification)	F	AATATGGACGTGGCTTAGCGT	60	77	[24]
		R	AGCCAAGCCTTGACGAACTAA			
blaZ	β-Lactamase (β-lactam resistance)	F	CAGTTCACATGCCAAAGAG	50	772	[27]
		R	TACACTCTTGGCGGTTTC			
mecA	PBP2a, methicillin resistance	F	GGGATCATAGCGTCATTATTC	61	527	[27]
		R	AACGATTGTGACACGATAGCC			
ermC	rRNA methylase (macrolide-lincosamide resistance)	F	ATCTTTGAAATCGGCTCAGG	48	295	[27]
		R	CAAACCCGTATTCCACGATT			
msrA	Macrolide efflux pump	F	GCTTAACATGGATGTGG	54	1230	[28]
		R	GATTGTCCTGTTAATTCCC			
aac(6′)-aph(2″)	Aminoglycoside modifying enzyme	F	CAGAGCCTTGGGAAGATGAAG	61	348	[25]
		R	CCTCGTGTAATTCATGTTCTGGC			
aph(3′)	Aminoglycoside phosphotransferase	F	CCGCTGCGTAAAAGATAC	57	610	[25]
		R	GTCATACCACTTGTCCGC			
tet(K)	Tetracycline efflux pump	F	TTAGGTGAAGGGTTAGGTCC	55	718	[27]
		R	GCAAACTCATTCCAGAAGCA			
tetM	Ribosomal protection protein (tetracycline resistance)	F	GTTAAATAGTGTTCTTGGAG	45	656	[27]
		R	CTAAGATATGGCTCTAACAA			
dfrG	Trimethoprim-resistant dihydrofolate reductase	F	TTTCTTTGATTGCTGCGATG	51	501	[27]
		R	AACGCACCCGTTAACTCAAT			
sea	Staphylococcal enterotoxin A	F	CCTTTGGAAACGGTTAAAACG	58	127	[29]
		R	TCTGAACCTTCCCATCAAAAAC			
seb	Staphylococcal enterotoxin B	F	GTATGGTGGTGTAACTGAGCA	53	351	[30]
		R	TCAATCTTCACATCTTTAGAATCA			
sec	Staphylococcal enterotoxin C	F	CTCAAGAACTAGACATAAAAGCTAGG	55	271	[30]
		R	TCAAAATCGGATTAACATTATCC			
sed	Staphylococcal enterotoxin D	F	CTAGTTTGGTAATATCTCCTTTAAACG	55	319	[30]
		R	TTAATGCTATATCTTATAGGGTAAACATC			
see	Staphylococcal enterotoxin E	F	CAGTACCTATAGATAAAGTTAAAACAAGC	55	178	[30]
		R	TAACTTACCGTGGACCCTTC			
seg	Staphylococcal enterotoxin G	F	AAGTAGACATTTTTGGCGTTCC	55	287	[30]
		R	AGAACCATCAAACTCGTATAGC			
sei	Staphylococcal enterotoxin I	F	GGTGATATTGGTGTAGGTAAC	55	454	[30]
		R	ATCCATATTCTTTGCCTTTACCAG			

F—forward primer; R—reverse primer; bp—amplicon size (base pairs); Ta—annealing temperature (°C); nuc—thermonuclease gene (*S. aureus* species marker); blaZ—β-lactamase gene; mecA—methicillin resistance gene encoding PBP2a; ermC—rRNA methylase gene (macrolide–lincosamide–streptogramin B resistance); msrA—macrolide efflux pump gene; aac(6′)-aph(2″)—aminoglycoside-modifying enzyme gene; aph(3′)—aminoglycoside phosphotransferase gene; tet(K)—tetracycline efflux pump gene; tetM—ribosomal protection protein gene (tetracycline resistance); dfrG—trimethoprim-resistant dihydrofolate reductase gene; sea–see—staphylococcal enterotoxin genes, types A–E; seg, sei—staphylococcal enterotoxin gene cluster (egc) genes.

**Table 2:** Prevalence of *S. aureus* and source-specific contamination risk.

Sample Source	*n* (total)	*n* (*S. aureus*+)	Rate (%)	OR	95% CI	*p*_corr_
Beef	67	5	7.5	0.58	0.19–1.63	1.000
Poultry meat	201	18	9	0.66	0.35–1.23	1.000
Horse meat	23	1	4.3	0.33	0.04–1.98	1.000
Pork	20	2	10	0.83	0.17–3.15	1.000
Meat products (sausages, frankfurters)	102	7	6.9	0.51	0.21–1.18	0.697
Raw cow’s milk	245	47	19.2	2.75	1.74–4.38	<0.001 *
Dairy products (cheese, cottage cheese)	53	4	7.5	0.59	0.18–1.76	1.000
Total	711	84	11.8	-	-	-

OR—odds ratio relative to all other sources; *p*_corr_—Bonferroni-corrected *p*-value. * Statistically significant after correction (*p*_corr_ < 0.05).

**Table 3:** Antimicrobial susceptibility profile of *S. aureus* isolates (n = 84).

Class	Antibiotic	R, n (%)	I, n (%)	S, n (%)
β-Lactams	Ampicillin	44 (52.4)	0 (0.0)	40 (47.6)
	Amoxicillin	13 (15.5)	17 (20.2)	54 (64.3)
	Benzylpenicillin	42 (50.0)	0 (0)	42 (50)
	Cefoperazone	4 (4.8)	18 (21.4)	62 (73.8)
	Cefoxitin	17 (20.2)	0 (0.0)	67 (79.8)
Tetracyclines	Tetracycline	34 (40.5)	0 (0)	50 (59.5)
	Doxycycline	16 (19.0)	9 (10.7)	59 (70.2)
Macrolides	Erythromycin	27 (32.1)	0 (0)	57 (67.9)
	Tylosin	22 (26.2)	10 (11.9)	52 (61.9)
Aminoglycosides	Streptomycin	0 (0.0)	0 (0.0)	84 (100)
	Kanamycin	3 (3.6)	8 (9.5)	73 (86.9)
	Neomycin	1 (1.2)	6 (7.1)	77 (91.7)
	Gentamicin	1 (1.2)	0 (0.0)	83 (98.8)
Sulfonamides	Sulfamethoxazole/trimethoprim	8 (9.5)	0 (0.0)	76 (90.5)
Fluoroquinolones	Ciprofloxacin	5 (6.0)	75 (89.3)	4 (4.8)
	Norfloxacin	7 (8.3)	0 (0.0)	77 (91.7)

R—resistant; I—susceptible at increased exposure; S—susceptible. Category I was not counted as resistance in MDR classification. For ciprofloxacin, isolates falling between the resistant breakpoint and the off-scale susceptible breakpoint were not interpreted as fluoroquinolone-resistant.

**Table 4:** Distribution of *S. aureus* isolates by number of antimicrobial agents to which resistance was detected (n = 84).

No. of Antibiotics (Resistance)	n	% (of n = 84)
0	21	25
1	13	15.5
2	12	14.3
3	14	16.7
4	8	9.5
≥5	16	19
Total	84	100

**Table 5:** Distribution of *S. aureus* isolates by number of antimicrobial classes (MDR).

Number of Resistant Antimicrobial Classes	n	% (of 84)
0 (susceptible)	21	25
1 class	29	34.5
2 classes	17	20.2
≥3 classes (MDR)	17	20.2
Total	84	100

**Table 6:** Distribution of phenotypic staphylococcal enterotoxin profiles (A–E) detected by ELISA among SE-positive *S. aureus* isolates (n = 22).

Phenotype (Toxin Combination)	n	% (of SE+)
E	4	18.2
D	3	13.6
A	2	9.1
C	2	9.1
D/E	4	18.2
A/B/C	2	9.1
A/B/C/D	1	4.5
A/B/E	1	4.5
A/B/C/D/E	3	13.6
Total (SE+)	22	100

**Table 7:** Frequency of antimicrobial resistance and virulence genes in *S. aureus* (n = 84).

Category	Gene	Function	n	% (of 84)
β-Lactams	*blaZ*	β-lactamase	37	44.0
	*mecA*	PBP2a (methicillin resistance)	4	4.8
Tetracyclines	*tet*(K)	Efflux pump	52	61.9
	*tetM*	Ribosomal protection	28	33.3
Macrolides	*ermC*	rRNA methylase	30	35.7
	*msrA*	Efflux pump	12	14.3
Aminoglycosides	*aac*(6′)-*aph*(2″)	Enzymatic modification	3	3.6
	*aph*(3′)	Phosphotransferase	3	3.6
Sulfonamides	*dfrG*	Dihydrofolate reductase	4	4.8
Enterotoxins	*sea*	Enterotoxin A	4	4.8
	*seb*	Enterotoxin B	1	1.2
	*sec*	Enterotoxin C	1	1.2
	*sed*	Enterotoxin D	0	0
	*see*	Enterotoxin E	1	1.2
	*seg*	Enterotoxin G (egc cluster)	8	9.5
	*sei*	Enterotoxin I (egc cluster)	8	9.5

**Table 8:** Genotypic–phenotypic concordance for antimicrobial resistance and toxigenicity markers in *S. aureus*.

Gene	Antibiotic/Toxin	Se (%)	Sp (%)	PPV (%)	NPV (%)	κ	Interpretation
*aph*(3′)	Kanamycin	66.7	98.8	66.7	98.8	0.65	Substantial
*blaZ*	Benzylpenicillin	76.2	88.1	86.5	78.7	0.64	Substantial
*tetM*	Tetracycline	70.6	92.0	85.7	82.1	0.64	Substantial
*sea*	SE type A	44.4	100	100	93.8	0.59	Moderate
*aac*(6′)-*aph*(2″)	Gentamicin	100	97.6	33.3	100	0.49	Moderate
*dfrG*	SMX/TMP	37.5	98.7	75.0	93.8	0.47	Moderate
*tet*(K)	Tetracycline	88.2	56.0	57.7	87.5	0.41	Moderate
*ermC*	Erythromycin	59.3	75.4	53.3	79.6	0.34	Fair
*mecA*	Cefoxitin	23.5	100	100	83.8	0.33	Fair
*msrA*	Erythromycin	25.9	91.2	58.3	72.2	0.20	Slight
*sec*	SE type C	12.5	100	100	91.6	0.21	Slight
*see*	SE type E	8.3	100	100	86.7	0.13	Slight
*seb*	SE type B	0	98.7	0	91.6	−0.02	Poor
*sed*	SE type D	0	100	N/A	86.9	0.00	Poor

Se—sensitivity; Sp—specificity; PPV—positive predictive value; NPV—negative predictive value; κ—Cohen’s kappa coefficient; SMX/TMP—sulfamethoxazole/trimethoprim; N/A—not applicable. Category I was not counted as resistance in MDR classification. For ciprofloxacin, isolates falling between the resistant breakpoint and the off-scale susceptible breakpoint were not interpreted as fluoroquinolone-resistant.

## Data Availability

The original contributions presented in this study are included in the article. Further inquiries can be directed to the corresponding authors.

## Supplementary Materials

The following supporting information can be downloaded at: https://www.mdpi.com/article/10.3390/vetsci13070687/s1, Table S1: Disk contents, manufacturers, interpretative standards, zone-diameter criteria, and quality-control ranges for antimicrobial susceptibility testing; Table S2: Isolate-level dataset for all 84 confirmed *S. aureus* isolates; Table S3: Raw data used to calculate sensitivity, specificity, positive predictive value (PPV), negative predictive value (NPV), and Cohen’s kappa coefficient.
